# Experiences of women with hypertensive disorders of pregnancy: a scoping review

**DOI:** 10.1186/s12884-022-04463-y

**Published:** 2022-02-22

**Authors:** Sachiko Sakurai, Eri Shishido, Shigeko Horiuchi

**Affiliations:** Department of Midwifery, Graduate School of Nursing Science, St Luke’s International University, 10-1 Akashi-cho, Chuo-ku, Tokyo, 1040044 Japan

**Keywords:** Qualitative research, Pre-eclampsia, Hypertension pregnancy-induced, Pregnancy, Postpartum period, Perception, Mothers, Delivery, Obstetric / psychology*, Hypertensive disorders of pregnancy

## Abstract

**Background:**

Hypertensive disorders of pregnancy (HDP) constitute one of the leading causes of maternal and perinatal mortality worldwide, and are associated with an increased risk of recurrence and future cardiovascular disease. HDP affect women’s health condition, mode of birth and timing, length of hospital stay, and relationship with their newborn and family, with future life repercussions.

**Aims:**

To explore the experiences of women with HDP from pregnancy to postpartum, and to identify (a) their perceptions and understanding of HDP, (b) their understanding of future health risks, and (c) the possible interventions by healthcare providers.

**Methods:**

A scoping review was conducted following the Joanna Briggs Institute method and in accordance with the PRISMA-ScR checklist. The following databases were searched from 1990 to 2020 (October): MEDLINE (PubMed), EMBASE, Cochrane Library, CINAHL, PsycINFO, and Google Scholar database. The Critical Appraisal Skills Programme (CASP) checklist was used as a guide for the qualitative analysis. Content analysis and synthesis of findings were conducted using Nvivo12.

**Results:**

Of the 1971 articles identified through database searching, 16 articles met the inclusion criteria. After data extraction, content analysis yielded six categories: ‘Life-threatening disorder’, ‘Coping with HDP’, ‘Concerns for baby and challenges of motherhood’, ‘Fear of recurrence and health problems’, ‘Necessity of social and spiritual support’, and ‘Positive and negative experiences in the healthcare context’. Women faced complex difficulties from the long treatment process while transitioning to motherhood.

**Conclusion:**

Our findings revealed the perceptions and understanding of women regarding HDP as a life-threatening disorder to both mothers and their babies which mothers need to cope with. Recovery of physical condition and the long-term psychological effects of HDP on women should be given attention by mothers and HCP to reduce future health risks. Importantly, a lifelong follow-up system is recommended for women with HDP.

**Supplementary Information:**

The online version contains supplementary material available at 10.1186/s12884-022-04463-y.

## Background

In 2015, The World Health Organization (WHO) set 17 global goals to be achieved by 2030, including goals to ‘reduce maternal mortality’ (Target 3.1) and ‘end preventable death of newborns and children under 5 years of age’ (Target 3.2) [1]. This implies the need to comprehensively understand the problems that contribute to maternal morbidity and mortality. Hypertensive disorders of pregnancy (HDP) are examples of such problems. This group of disorders is related to hypertension occurring before pregnancy, during the first 20 weeks of pregnancy, or after 20 weeks of pregnancy such as gestational hypertension and preeclampsia including the more severe subtypes eclampsia and hemolysis, elevated liver enzymes and low platelets’ (HELLP) syndrome [2, 3]. These HDP constitute one of the leading causes of maternal mortality worldwide, particularly in low-income countries. Hypertensive disorders are a leading cause of maternal mortality in Latin America and the Caribbean (26%), Asia and Africa (9%), and in developed countries (16%) [4]. The worldwide prevalence rate of preeclampsia is in the range of 2-10% of pregnancies [5]. The risks associated with HDP are fetal growth restriction, placenta abruption, preterm delivery, and cesarean section [6]. Additionally, preeclampsia increases the risk of fetal mortality, stillbirth, premature birth, and low birth weight [7].

Numerous researchers have studied the management, treatment, and control of HDP, with recommendations published by organizations such as the International Society for the Study of Hypertension in Pregnancy (ISSHP) [3], American College of Obstetricians and Gynecologists [8], and WHO [9]. There are also recommendations for non-pharmacological interventions by ISSHP such as exercise to maintain the appropriate body weight [3]. Post-discharge follow-up for hypertension was reportedly performed within 3-14 days according to the postpartum medication, noting the risks of preeclampsia and eclampsia during the postpartum period [6]. Furthermore, a three-month follow-up for physical conditions including blood pressure, and a lifelong follow-up for cardiovascular disease (CVD) risk were recommended by ISSHP [3].

Evidence suggests that HDP carries a risk of long-term health sequalae for women [3]. Prevention of HDP complications reduces not only women’s mortality but also their negative experiences with preeclampsia. Women with preeclampsia may have a life-threatening experience, premature birth, and long hospitalization. They are affected by fear, disrupted relationship with their newborns, and separation from loved ones [10]. Additionally, preeclampsia and gestational hypertension were found to have an association with increased risk of recurrence [6, 11] and future CVD [12, 13], and Type 2 diabetes [14]. HDP affect not only the mother’s health condition but also her timing and mode of birth, length of hospital stay [6], and relationship with the newborn and family [10], which extends into their later life.

Although there have been advanced medical approaches, reducing maternal mortality and improving maternal care remain big challenges. Importantly, the narrative experiences of mothers can reflect the interventions and care administered by healthcare providers (HCP), as well as their needs and support requirements. A previous systematic review of women’s views on intrapartum care revealed their values and expectations for HCP, as well as their directions on what should be kept in mind [15]. Women’s voices and experiences can provide a better understanding of their conditions and perceptions of their own disease. This information could be used to make health counselling more effective for women who are at high-risk during pregnancy [16]. Therefore, the experiences of women with HDP are explored in this study.

## Aims

This study aimed to explore the experiences of women with HDP from pregnancy to postpartum, and to identify (a) their perceptions and understanding of HDP, (b) their understanding of future health risks, and (c) the possible interventions including mutual relationship and care by healthcare providers for improving health.

## Methods

We conducted a scoping review to map, synthesize, and summarize research evidence to vividly convey the experiences of women with HDP [17, 18]. The Johanna Briggs Institute and Preferred Reporting Items for Systematic Reviews and Meta-Analyses extension for Scoping Reviews (PRISMA-ScR) checklists [19] were used as methodological guides. The Critical Appraisals Skills Programme (CASP) checklist was used for qualitative analysis [20].

### Inclusion criteria

The inclusion criteria were based on questions using the Population, Concept, and Context (PCC) framework indicated in the Johanna Briggs Institute guide [19] and as shown in Additional File 1. The participants were women diagnosed with HDP such as a) preeclampsia-eclampsia, b) chronic hypertension (of any cause), c) chronic hypertension with superimposed preeclampsia, and d) gestational hypertension [2] during pregnancy to the postpartum period. Articles describing the experiences of women with HDP at any health facilities in any country were included. Specifically, studies were included if they were original research with either qualitative or mixed methods study design published in the English language only. The aim was to capture the lived experiences of women with HDP to reflect their voices. The publication year was set from 1990 to October 2020.

### Search strategy

We searched MEDLINE (PubMed), EMBASE, Cochrane Library, CINAHL, and PsycINFO. Relevant articles were also added from the hand search of Google Scholar. The keywords were developed from the PCC framework. The reference lists of included articles were hand-searched to identify other key articles. The complete search strategy for MEDLINE (PubMed) is shown in Additional File 2**.**

### Article selection

There were 1971 articles retrieved from the database searches; 1966 articles were from database searches and five articles were hand-searched from the reference lists. After the duplicates were removed, the titles and abstracts were screened based on the inclusion criteria. Then, 27 full-text articles were read of which 11 articles were excluded (Additional File 3). These articles were excluded because in six articles, women with HDP were not specifically identified among the study population of women with high-risk pregnancies. In one article, the participants were not identified as having HDP. In two articles, the aims of the studies were different, which were blood pressure self-management and primary prevention of HDP. Finally, two papers were not full articles; one was a conference abstract and the other was an ePoster. Two reviewers independently reviewed and discussed the eligibility of the articles for inclusion. If disagreements could not be resolved through discussion, a third reviewer made the decision about the selection. Finally, 16 articles were included in the qualitative synthesis.

### Data extraction

Data were extracted based on the three study aims using the PCC framework which includes the author, year of publication, country of origin, aims, participants, and key findings as indicated in the Johanna Briggs Institute guide [19]. Content analysis was conducted to identify similarities and differences among retrieved categories and themes of the studies using Nvivo12 software. Two reviewers read all the articles several times to understand the main concepts.

### Quality assessment

The two reviewers assessed the quality of the articles using the CASP checklist [20]. This checklist is a tool for systematically screening articles through enquiry and critique of their validity, results, and research contributions.

## Results

**Fig. 1:**
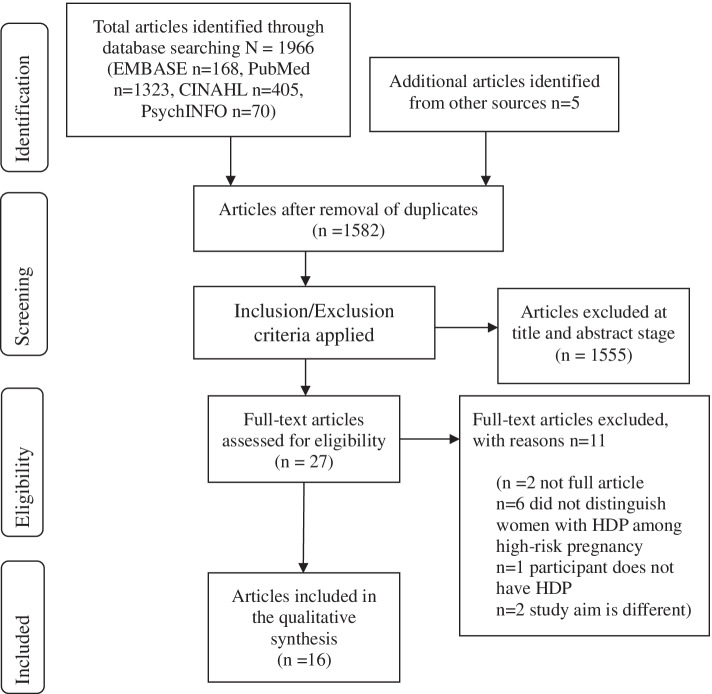
PRISMA flowchart of article screening and selection process

Of the 1971 articles identified through database searching, 16 articles met the inclusion criteria. The PRISMA flowchart of article screening and selection process is shown in Fig. 1. A summary of the findings of each study is shown in Additional File 4.

Of these 16 articles, eight from low- and middle-income countries (one from Nigeria [21], one from Tanzania [22], one from Rwanda [23], one from South Africa [24], and four from Brazil [25–28]) and 8 from high-income countries (four from the UK [29–32], two from Norway [33, 34], one from Australia [35], and one from the USA [36]). These studies include six semi-structured interviews [22, 27, 29, 30, 32, 35], four in-depth interviews [21, 23, 24, 31], two interviews [34, 36], two focus group interviews [28, 33], one word association technique [26], and one combination of semi-structured interviews and word association test [25]. The interviews were conducted during the postpartum period in most articles, during the pregnancy period in two articles [29, 32], during both pregnancy and postpartum periods in one article [25], and during an extended period of 13 years between diagnosis and interview in two articles [31, 36].

The participants’ diagnoses were preeclampsia (*n* = 10) [23–25, 27, 28, 30, 31, 33, 34, 36], eclampsia (*n* = 1) [22], both (*n* = 1) [21], gestational hypertension or preeclampsia (*n* = 1) [35], hypertension in pregnancy (*n* = 2) [29, 32], and HDP (*n* = 1) [26]. In three articles, the participants had infants hospitalized in the neonatal intensive care unit (NICU) or born prematurely [26, 28, 34], and two participants were women admitted to the intensive care unit (ICU) [22, 24].

### Categories and subcategories of extracted data

Six categories were synthesized from the extracted data: ‘Life-threatening disorder’, ‘Coping with HDP’, ‘Concerns for baby and challenges of motherhood’, ‘Fear of recurrence and health problems’, ‘Necessity of social and spiritual support’, and ‘Positive and negative experiences in the healthcare context’. The articles supporting these categories are summarized in Additional File 5.

The four major components that were integrated with the lived experiences of women with HDP were as follows: Mother, Child (Concerns for the baby), Social support, and Healthcare. The trajectory of the lived experiences of women with HDP is shown in Additional File 6.

The trajectory of the lived experiences of women started with the diagnosis of HDP as a life-threatening disorder that appeared to rapidly change their daily life and understanding about themselves into a new lifestyle. The lived experiences had emotional and physical impacts. The two aspects of the lived experiences were coping with HDP and concerns for their baby and challenges of motherhood. The conflicts inherent in a life-threatening disorder became embodied in a mother attempting to raise her child. Women had to cope and struggle with the changing situation they faced. Social and spiritual support became very important. Sufficient information, adequate skills of HCP, and continuity of care led to women’s positive experiences. A demand for better healthcare emanated from women’s perceived lack of information about HDP and objectionable HCP attitudes.

Mother, Child (Concerns for baby), Social support, and Healthcare were the four major components upon which the women’s experiences were constructed. The perceptions and experiences of women changed over time, and were influenced by the women’s health condition, treatment, birth, and child-bearing. The women’s perceptions and experiences were synthesized into six categories with supporting subcategories, and the four major components of their lived experiences were intertwined into a comprehensive overview of their lived experience.

### Category 1: life-threatening disorder

This category included four subcategories: *Death is a real possibility; Anguish, blame, and seeking answers; Readiness to accept HDP;* and *Unexpected birth experiences*. Because of the impact of HDP diagnosis, women were confused and emotionally overwhelmed, and had to undergo an earlier birth than they expected or wanted. Their reactions varied based on their awareness of HDP from their previous experience or family history.

#### Death is a real possibility

HDP were perceived as life-threatening to the mother and her infant. Most articles noted that women perceived the risk of dying, stroke, or death because of their HDP [21, 23–25, 28, 31, 34–36]. The word association test revealed that death was a stronger representation of HDP [26]. Also, the risk of stillbirth created a fear of fetal death in utero [21, 25, 27, 28, 31, 34–36].

#### Anguish, blame, and seeking answers

Because of the life-threatening risk of HDP, there was a sudden change in women’s daily life. Women were taken by surprise, and they recognized that they are ‘not normal’ [27, 32, 35, 36]. Women felt condemned by the community for not functioning normally [23]. HDP diagnosis caused various emotions such as “anguish, doubt, sadness, despair, difficulties, surprises, escape and blame” [26]. Other articles reported “anguish, agony, anxiety, madness, fear, consternation, and panic” [25, 27, 36]. Women attempted to determine the causes or questioned their understanding after the diagnosis [23, 27, 28, 30, 32, 33, 35]. They considered their lifestyle, stress, or life events as possible causes [29]. Women without symptoms had a more difficult time in understanding their situation [29, 32].

#### Readiness to accept HDP

The impact of HDP was influenced by women’s readiness to accept HDP. Women were not surprised by the diagnosis if they have a family history or previous experience of HDP [21, 32, 35], which helped them understand the disease [21, 29]. Previous experiences drove women to seek earlier antenatal clinic attendance [21]. By contrast, women were unaware of HDP during their antenatal care in Brazil [28] and had scarce knowledge of HDP in Rwanda [23]. In Nigeria, women considered HDP as being caused by stress or spiritual interference [21].

#### Unexpected birth experience

Because of HDP, women suddenly had to face an earlier birth. This birth experience was unexpected [27, 34], different from what they had planned, and did not conform to the normal pregnancy period [31, 33, 35, 36]. One article described women’s paradoxical feelings of relief from bodily discomfort and shock from their sudden birth [34].

### Category 2: coping with HDP

After an HDP diagnosis, women struggled with their situation during admission and even after discharge as it changed rapidly depending on their health condition, treatment, and physical symptoms. Women were left to handle their succession of difficulties. Coping with HDP had three subcategories: *Loss of control; Coping with various physical symptoms;* and *Struggling with the prolonged treatment process: Mother and child.*

#### Loss of control

Women experienced health emergencies and felt loss of control of their situation and became dependent on healthcare [22, 27, 31, 34, 35]. Women experienced seizures and different treatments in the ICU, thus they felt like dreaming [22] or delusional [24].

#### Coping with various physical symptoms

Seven studies [22, 24, 26, 29, 31, 34, 36] focused on women’s recall of various physical symptoms, which included severe headache, abdominal pain before seizure [22], painful edema, stomachache or headache [32, 34], swelling, overall sickness [29, 36], fatigue [32, 36], and dizziness [34]. Women sought pain relief and improvement of sleep [31].

#### Struggling with the prolonged treatment process: mother and child

Birth was described as a cure for the HDP, but it was also the beginning of the long road to recovery which deprived mothers of their daily activities [31]. Mothers who survived showed appreciation [24, 34, 35]. Some mothers longed to go home early to resume their usual life activities with their baby and family [28, 35]. Women needed time to realize and accept their situation [34], and to move forward [35]. Moreover, trauma or psychological problems persisted after discharge into the postpartum period [24, 31, 33].

### Category 3: concerns for baby and challenges of motherhood

The bonding between mothers and their child started before the HDP diagnosis. Mothers showed a strong attachment towards their baby during treatment. After birth, women tried their best to care for their premature infant who was separated from them despite some going through a life-threatening disorder. This category has three subcategories: *Fears about baby during pregnancy; Emotional roller coaster: Premature baby in NICU;* and *Facing bonding obstacles*.

#### Fears about baby during pregnancy

Mothers feared that HDP would affect their baby’s health and development, cause problems after birth [25, 27, 31, 35], or result in a premature baby [21, 27, 34]. Mothers worried that they might die early leaving their infant alone [23, 27, 35].

#### Emotional roller coaster: premature baby in NICU

A consequence of HDP treatment was preterm birth. After birth, mothers feared that their baby may die or not continue normal development [26, 27, 31, 34]. NICU treatment involving medical devices created feelings of guilt among mothers [26, 34]. Other feelings included “shock, sadness, insecurity, despair, agitation, [as well as] joy and happiness” [28].

#### Facing bonding obstacles

Babies were separated because of their prematurity or the mother’s seizure. Longing for and not being with their infant were difficult [22, 27, 31, 34, 35]. Thus, the condition of the mother or baby meant loss of early attachment after the birth experience [22, 31, 33, 35]. This delayed mothers from establishing early bonding with their babies [31, 34, 35]. In addition, mothers experienced obstructions caused by NICU medical devices [27, 28, 34]. The physical exhaustion or symptoms of mothers became obstacles to being with their baby [22, 34]. Although they had difficulty in bonding, mothers devoted care to their babies as a priority [22, 27], and they felt delighted with the interactions with their babies [27, 28, 35].

### Category 4: fear of recurrence and health problems

Some women were aware of the risk of HDP recurrence and its effect on their future health. They sought ways to prevent HDP according to their capabilities. This category has three subcategories: *Avoiding next pregnancy; Attending to future health issues;* and *Attempting preventive measures.*

#### Avoiding next pregnancy

Women feared HDP recurrence in their future pregnancies [22, 25, 27, 30, 35]. Some expressed avoiding another pregnancy [22, 30, 33] even though they wanted another child [27]. Some planned to have another baby [30] as they assumed that the next pregnancy would be monitored more closely [35].

#### Attending to future health issues

Some women worried that if HDP recurred, their cesarian section scars might become a problem [22]. Some doubted if they had fully recovered from their HDP [23]. Women were also concerned of their child’s future health [31]. They were aware of future health problems such as CVD particularly when they have a family history of HDP [30].

#### Attempting preventive measures

Although women feared the possibility of HDP in the future, they also sought ways to prevent HDP [22] such as changing their diet [25, 30, 33], exercise [30], stress management [33], and “taking responsibility of their own health” [30].

### Category 5: necessity of social and spiritual support

Social and spiritual support was very important for women throughout the trajectory from HDP diagnosis to postpartum. Without support, women might not have been be able endure their situation. Support came from their husbands/partners, family members (mother, father, mother-in-law, sister), friends, community members, and God. This category has two subcategories: *Stronger social networks* and *Belief in God.*

#### Stronger social networks

Eight articles mentioned the support received from a husband/partner and other family members. Husbands played a crucial role for women who were conflicted by their situation from the time of HDP diagnosis to delivery, and during the postpartum period [21, 23, 24, 27, 29, 30, 35, 36]. The need for social support increased during pregnancy [36] and when staying in the hospital with a premature baby [35]. With social support, women became more stable emotionally and felt safe during childbirth [27].

Husbands and family members influenced women to seek healthcare [21]. In Nigeria, traditional attendants and herbalists provided support, although they might have delayed women’s necessary healthcare [21]. Peers of mothers who had labor ward birth or cesarian section influenced women by either giving reassurance or causing anxiety [29].

#### Belief in god

Two aspects of relationship with God have been identified: “grateful to God” and “asking for God’s help” [22]. Women showed appreciation for being helped and surviving [21–24], and resolve to overcome further difficulties [22–24], especially those who lost their baby [22]. In some instance, the church could not meet the women’s needs [36].

### Category 6: positive and negative experiences in the healthcare context

Most articles discussed women’s antenatal and postpartum healthcare. There were areas where women were satisfied with their healthcare. There were also situations where women were offended by the attitude or inadequate explanation of HCP. This created a demand for better care. Although medical services were available, some women could not avail of them because of obstacles. This category has three subcategories: *Development of trust; Demand for better healthcare;* and *Specific obstacles to healthcare.*

#### Development of trust

Women felt reassured when receiving specialized antenatal care [32], in-hospital care [35], and physical care (e.g., bed bath or feeding in ICU) [22]. Contributing to perceptions of positive care were well-informed HCP, trust in the expertise of HCP [21, 29, 35], and continuity of care [21, 35]. Regarding the care provided to their baby in the NICU, mothers expressed satisfaction [28] and appreciation for professional qualifications [26]. HCP also played an important role in the absence of a partner’s support [27].

#### Demand for better healthcare

Studies have pointed to the lack of information provided or the insufficient and inadequate explanation about HDP or what had happened to the women [21–23, 25–27, 32, 33, 36] and to their babies [28, 22], including extent to future health situations [30, 33]. However, women were reportedly oblivious to their eclampsia [22].

Women often discussed the attitudes or communication styles of HCP. Women were stressed by not being given sufficient attention [23, 36] and the lack of coherence between staff members [29, 36] with HCP excessively concentrating on the care of their baby in the NICU and failing to notice the care needs of mothers [28]. Some women found that their clinicians trivialized their HDP diagnoses, which delayed adequate care [33], or that the information provided was not evidence-based [36]. The interventions of HCP elicited uncertainty and anxiety in some women [22]. During the postpartum period, the demand for follow-up by HCP was also reported [30].

#### Specific obstacles to healthcare

Financial burden or lack of insurance prevented some women from availing of the most qualified care in Rwanda [23] and Nigeria [21]. In Nigeria, distance to the facility was a barrier for women. Even more distressing was the disrespectful attitude amounting to abuse by HCP commonly perceived among women as “mistrust” for healthcare [21].

## Discussion

The findings revealed that HDP were perceived as life-threatening disorders for mothers and their babies, necessitating mothers to cope with the situation of loss of control and being overwhelmed. Interactions with HCP had both positive and negative effects on women. Some women felt assured and well-informed by the care provided by HCP. At the same time, women needed more information and were occasionally disappointed with the attitudes of HCP and how they communicated. Even after overcoming HDP, some mothers experienced fear of recurrence and future health problems as revealed in this scoping review.

This scoping review of the experiences of women with HDP also revealed the long-term effects of HDP on their mental and physical health in the context of future risks. The recall of bad memories included life-threatening and overwhelming experiences that persist for a long time among the women.

### Regional differences and prevailing problems in healthcare

From the women’s viewpoint, their voices reflected their needs for healthcare. Access to healthcare might be influenced by the country’s political system or economic development as experienced in Nigeria [21] and Rwanda [23]. Meta-analysis of risk factors for preeclampsia and eclampsia in Sub-Saharan Africa showed that having no antenatal care visits increased the risk of preeclampsia and eclampsia by nearly threefold [37]. In contrast, the weaknesses of healthcare such as lack of information and inadequate explanations were not differentiated among countries. These are the prevailing challenges irrespective of a country’s background. Women needed appropriate information, otherwise they questioned the assessment and management skills of HCP [33]. There were instances wherein HCP lacked knowledge about the diagnosis [38], prevention and management [39] of preeclampsia. HCP have a responsibility to know the latest information, use optimal management strategies, improve their skills, and acquire knowledge pertinent to the women’s condition.

### Empowerment of women

Although women with HDP suffer and struggle similarly to previous findings [10], the positive events expressed by women should also be emphasized. Interactions with their babies facilitated joy or happiness. Some women initially perceived that their babies belonged to the hospital, then gradually they claimed their babies as their own [34]. The attitude of HCP in the NICU is important; thus, a policy of open access, family involvement, and environmental and psychological support has been recommended [40]. Trust with healthcare or the continuity of care [21, 35] supported women through better management of complications. Continuity of care improved clinical outcome and satisfaction, built trust between midwives and women, increased personalized care, and empowered women [41].

### Long-term effects of HDP on women

Our review elucidated the long-term effects of HDP on women. Their voices revealed the persistence of trauma or psychological problems after hospital discharge [24, 31, 33]. Life-threatening and uncontrollable experiences had a strong impact on the mental health of women. Although the psychological condition of women recovered with time, 25% of women continued to have a poor psychological condition 1 year after giving birth and had an earlier gestational term [42]. A narrative review of 17 studies revealed increased prevalence and severity of depression, anxiety, and posttraumatic stress disorder (PTSD) after overcoming HDP, although the associations among the studies were inconsistent [43]. Notably, treatment of physical symptoms is not the goal of care for women with HDP.

Our review also showed that women suffered from a fear of HDP recurrence and future health complications. Such fear should also be followed up by HCP. As a result, women expressed their preference of not having another pregnancy [22, 30, 33] for two reasons: suffering from illness and prolonged hospitalization and treatment. The recurrence risk of HDP was found to range from 2 to 55% in subsequent pregnancies, depending on the previous diagnosis of either preeclampsia or gestational hypertension and gestation weeks before birth [6]. Women who became pregnant within a year had a better psychological condition than women who were not willing to become pregnant because their experience [42]. In fact, fear of HDP recurrence influenced the next pregnancy.

Thus, HDP affected the lifelong physical and mental health conditions of women. HCP should be aware of such effects and take initiative to learn more about HDP. As obstetrics appears to be focused on women from pregnancy to postpartum, the continuity of women’s healthcare after the postpartum period should not be disrupted. A follow-up system for sustaining women’s lifelong health through sharing of information and continuity of care should be considered.

### Strengths and limitations

In terms of strengths, this study complements previous reviews of the experiences of women with preeclampsia [10] and clarified the trajectory of experiences of women with HDP from pregnancy to the postpartum period. Moreover, this study not only revealed rapidly changing emotional and physical responses as well as interactions with babies and HCP, but also clarified women’s concerns about the risks of HDP recurrence and future health problems that they might have to cope with and the psychological effects. The overview of such experiences would give HCP better insights and would broaden their understanding when interacting with HDP patients. These aspects also indicate that the disappearance of symptoms and the completion of treatment do not necessarily mean an end to the care for women with HDP. This scoping review emphasizes the importance of long-term follow-up based on women’s voices. The rigor of this scoping review was enhanced using a methodology that followed the JBI guidelines for scoping review with a comprehensive search strategy selection by two reviewers.

On the other hand, the present study also has several limitations. In this scoping review, we included studies that explored the experiences of women with HDP. These studies covered from low- and middle-income to high-income countries. However, the present study lacked the experiences of women from Asian countries which have different social, cultural, and medical system backgrounds. The period of data collection which was mostly by interview ranged from pregnancy to 48 h postpartum and up to 13 years after giving birth. Four studies included women whose duration between interview and birth was more than 1 year [23, 31, 33, 36]. The recall of their memory might have been influenced by the situations they went through. This indicates that the overwhelming experience remained for a long time. Although social support was perceived as essential for women with HDP, the present review did not include the experiences of family members. As the family members may influence a woman’s healthcare-seeking decision [21], their perceptions might provide other insights on how to support women with HDP.

### Relevance to clinical practice

HCP should understand the significance of life-threatening HDP experiences and their effects on women’s health and changes in their daily life. It is crucial to identify the support needed by women based on their needs such as adequate and sufficient information and care. To best learn from women’s experiences and apply changes, a follow-up system for women’s lifelong healthcare should be considered to address the long-term effects of HDP on women’s mental and physical health. System revision involves a large-scale strategy of building a linkage between different healthcare institutions and task-sharing [44]. Continuity of access to health service would also improve the care for women.

## Conclusions

Our findings from the experiences of women with HDP revealed their perceptions of HDP as a life-threatening disorder to both mothers and their babies which mothers need to adjust to. Previous HDP experiences caused fear of HDP recurrence and future complications. Interactions with HCP affect women positively and negatively. Additionally, recovery of the physical condition and long-term psychological impact on women deserve optimal attention by HCP. It is important to not only address HDP treatment but also provide long-term psychological support from HCP. A lifelong follow-up system is essential and recommended for women with HDP.

## Supplementary Information

**Additional file 1.**

**Additional file 2.**

**Additional file 3.**

**Additional file 4.**

**Additional file 5.**

**Additional file 6.**

## Data Availability

Data sharing is not applicable to this article as no datasets were generated or analyzed during the current study.

## Abbreviations

WHO: World Health Organization
HCP: Healthcare providers
HDP: Hypertensive disorders of pregnancy
ISSHP: International Society for the Study of Hypertension in Pregnancy
PTSD: Posttraumatic stress disorder
PRISMA: Preferred Reporting Items for Systematic Reviews and Meta-Analyses
PRISMA-ScR: Preferred Reporting Items for Systematic Reviews and Meta-Analyses extension for Scoping Reviews
CASP: Critical Appraisal Skills Programme
CVD: Cardiovascular disease
