# Supplementary material for: Experiences of women with hypertensive disorders of pregnancy: a scoping review
Source: BMC Pregnancy Childbirth. 2022 Feb 22;22:146. doi: 10.1186/s12884-022-04463-y (PMC8864783; doi:10.1186/s12884-022-04463-y)
Supplement: Supplementary file 1 — Additional file 1. [file 12884_2022_4463_MOESM1_ESM.docx]

**Appendix A** Inclusion and exclusion criteria based on the population concept context (PCC) framework

| **PCC Framework** | **Inclusion Criteria** | **Exclusion Criteria** |
| --- | --- | --- |
| **Population** | Women diagnosed with  hypertensive disorders  of pregnancy |  |
| **Concept** | Experience, perception |  |
| **Context** | Any institutions and health  facilities from any other countries |  |
| Language | English | Non-English studies |
| Publication type  Qualitative studies | Full text available  Original studies | Only abstract is available (e.g., conference, reviews, trial registrations) |
| Publication date | 1990-2020 | Before 1990 |
