# Supplementary material for: Experiences of women with hypertensive disorders of pregnancy: a scoping review
Source: BMC Pregnancy Childbirth. 2022 Feb 22;22:146. doi: 10.1186/s12884-022-04463-y (PMC8864783; doi:10.1186/s12884-022-04463-y)
Supplement: Supplementary file 2 — Additional file 2. [file 12884_2022_4463_MOESM2_ESM.docx]

# **Appendix B** Search Strategy for MEDLINE (PubMed)

| #1 | eclampsia"[All Fields] OR "eclampsias"[All Fields] |
| --- | --- |
| #2 | preeclampsia"[MeSH Terms] OR "preeclampsia"[All Fields] OR "preeclampsia"[All Fields] |
| #3 | hypertension, pregnancy induced"[MeSH Terms] OR ("hypertension"[All Fields] AND "pregnancy induced"[All Fields]) OR "pregnancy-induced hypertension"[All Fields] |
| #4 | gestational"[All Fields] AND "hypertension"[All Fields]) OR "gestational hypertension"[All Fields] |
| #5 | hypertension, pregnancy induced"[MeSH Terms] OR ("hypertension"[All Fields] AND "pregnancy induced"[All Fields]) OR "pregnancy-induced hypertension"[All Fields] OR ("pregnancy"[All Fields] AND "induced"[All Fields] AND "hypertension"[All Fields]) OR "pregnancy induced hypertension"[All Fields] OR (("hypertense"[All Fields] OR "hypertension"[MeSH Terms] OR "hypertension"[All Fields] OR "hypertension s"[All Fields] OR "hypertensions"[All Fields] OR "hypertensive"[All Fields] OR "hypertensive s"[All Fields] OR "hypertensives"[All Fields]) AND ("disease"[MeSH Terms] OR "disease"[All Fields] OR "disorder"[All Fields] OR "disorders"[All Fields] OR "disorder s"[All Fields] OR "disordes"[All Fields]) AND ("pregnancy"[MeSH Terms] OR "pregnancy"[All Fields] OR "pregnancies"[All Fields] OR "pregnancy s"[All Fields] |
| #6 | #1 or #2 or #3 or #4 or #5 |
| #7 | percept"[All Fields] OR "perceptibility"[All Fields] OR "perceptible"[All Fields] OR "perception"[MeSH Terms] OR "perception"[All Fields] OR "perceptions"[All Fields] OR "perceptional"[All Fields] OR "perceptive"[All Fields] OR "perceptiveness"[All Fields] OR "percepts"[All Fields] |
| #8 | ("experience"[All Fields] OR "experience s"[All Fields] OR "experiences"[All Fields] |
| #9 | #7 or #8 |
| #10 | #6 and #9 |
| #11 | Filters: from 1990-2020, English |
|  | 1323 |
