# Supplementary material for: Experiences of women with hypertensive disorders of pregnancy: a scoping review
Source: BMC Pregnancy Childbirth. 2022 Feb 22;22:146. doi: 10.1186/s12884-022-04463-y (PMC8864783; doi:10.1186/s12884-022-04463-y)
Supplement: Supplementary file 3 — Additional file 3. [file 12884_2022_4463_MOESM3_ESM.docx]

# **Appendix C** Excluded articles

| **Article** | **Reason for exclusion** |
| --- | --- |
| Cowan (2004) | Not a full article (conference abstract) |
| Mapp (2005） | No clear distinction of patient category for HDP in the results |
| Mapp and Hudson (2005) | No clear distinction of patient category for HDP in the results |
| Markovic et al. (2006) | No raw data provided for HDP and no clear distinction of HDP in the results |
| Rööst et al. (2009) | No clear distinction of patient categories for HDP among a  life-threatening pregnancy-related complication |
| Meaney et al. (2016) | No raw data provided for HDP and no clear distinction of HDP in the results |
| Crombag et al. (2017) | Participants are not identified as having HDP |
| Fenn et al. (2019) | Not a full article (ePoster) |
| Nagraj et al. (2019) | No clear distinction of HDP patient category |
| Vestering et al. (2019) | Aim of study is different (primary prevention of HDP) |
| Cairns et al. (2020) | Aim of study is different (blood pressure self-management) |
