# Supplementary material for: Experiences of women with hypertensive disorders of pregnancy: a scoping review
Source: BMC Pregnancy Childbirth. 2022 Feb 22;22:146. doi: 10.1186/s12884-022-04463-y (PMC8864783; doi:10.1186/s12884-022-04463-y)
Supplement: Supplementary file 4 — Additional file 4. [file 12884_2022_4463_MOESM4_ESM.docx]

**Appendix D** Summary of screening results

| **Authors and Year** | **Country** | **Study Design** | **Data Collection　Method** | **Aim** | **Population** | **Key Findings** | **Relevant CASP Score/ Possible Total** |
| --- | --- | --- | --- | --- | --- | --- | --- |
| Jackson et al. (2006) | UK | Qualitative interview study | Semi-structured interview | To explore  　women’s views  　on being referred  to and attending a  　specialist  antenatal  　hypertension clinic | 21 women  referred to  pregnancy  hypertension  clinic | Described *Being referred* as  identification of ‘riskiness',  *Attending the clinic* as  reassurance, and  *Negotiating*  *normality*. | 10/10 |
| Lima de Souza et al. (2007) | Brazil | Qualitative | Focus group interview | To analyze maternal  experiences of  preeclampsia  pregnancy with  premature birth at  a neonatal  intensive care unit  (NICU) | 28 women with  preeclampsia  whose child  were  hospitalized in  NICU at state  facility Interviewed at  postpartum  period | Three thematic nuclei that  gave rise to nine categories,  23 subcategories, and 318  analysis units  These showed the  subjects’ lack of knowledge  regarding preeclampsia  and its association with  prematurity.  Difficulties inherent to the  maternal role of caring for  the child in NICU were  identified, accentuated by  communication flaws  between health  professionals and users. | 10/10 |
| Barlow et al. (2008) | UK | Exploratory cross-sectional, qualitative, descriptive study | Semi-structured interview | To document  women’s  experiences of  admission to  hospital with a  pregnancy-related  complication,  hypertension,  from their own  perspective | 12 women with  hypertensive  disease who  were admitted  to a university  hospital Interviewed  between one  and three  days after  hospital  admission | Summarized four  categories: Search for  meaning; Attribution of  causality; Information  needs; Social factors | 10/10 |
| Fleury et al. (2010) | Brazil | Qualitative, exploratory study | Semi-structured interviews | To assess the  development of  mother-child  relationship in  primiparous  women diagnosed  with preeclampsia  in the third  trimester of  pregnancy | 15 primiparous  women  diagnosed with  preeclampsia  with the baby  one to four  months old  at a tertiary  referral  hospital | All participants referred to  signs of primary maternal  preoccupation, the  importance of their  relationship with their own  mother as constituting a  model of childcare, the  relevance of social support,  and the difficulties  encountered in fully  understanding the medical  implications of the diagnosis  in their lives and in dealing  with hospitalization. Despite  feeling the effect of the  disease on their lives, these  women developed a good  mother–child relationship. | 10/10 |
| de Azevedo et al. (2011) | Brazil | Qualitative : Interview | Word association test,  Semi-structured interview | To understand the  meanings of  preeclampsia for  pregnant and  postpartum  women and  healthcare  professionals | 51 of pregnant  and 10  postpartum  women in  word  association  test,  18 pregnant and  two postpartum  women in  interview at  referral  hospital | Meaning of preeclampsia was  fear, risk, care, and lack of  information from word  association test. From the interview, this fear  was related to three main  aspects: Possibility of  dying or losing the infant;  Risk of both having  Diseases; Higher  probability of the mother  having preeclampsia in  future pregnancy. | 10/10 |
| Herbest et al. (2012) | South Africa | Qualitative case study | In-depth　Interview | To explore one  woman’s narrative  of awareness,  emotions and  thoughts during  treatment in an  intensive care unit  (ICU) | One woman with  preeclampsia  interviewed  after discharge  from ICU until  six months after  discharge from  hospital | Patients in ICU struggled with  delusions, helplessness,  mortality, loneliness,  isolation, and anxiety, but  may have also experienced  hope, faith, inner strength  and social support. | 10/10 |
| de Souza et al. (2013) | Brazil | Exploratory, descriptive and qualitative study | Free word association technique | To identify the  significance of  attributing  pregnant mothers  to hypertensive  disorders of  pregnancy (HDP),  and the  consequences,  such as premature  births and child  hospitalization in  the NICU | 70 women with  HDP who  visited the  NICU at least  two times  at a university  referral  hospital | The 1007 obtained evocations  constituted three thematic  units: Representation of  HDP, Prematurity, and  NICU. The categories death  and negative aspects were  inherent to the three units  analyzed, followed by  coping strategies and needs  for care present in HDP  and prematurity. | 8/10 |
| Brown et al. (2013) | UK | Qualitative | Semi-structured interview | To elicit women’s  personal  understanding of  future  cardiovascular  risk,  following  a pregnancy  complicated by  preeclampsia, and  to identify the  postnatal needs of  these women | 12 women with  preeclampsia  at national  hospital Interviewed at 24-  62 weeks  postpartum | Findings were categorized as  Follows: Women’s  understanding of risks to  future pregnancies;  Women’s awareness of  future cardiovascular risks;  Factors affecting women’s  experiences of the postnatal  clinic; Post-pregnancy  perspectives on health and  information needs | 10/10 |
| Kehler et al. (2016) | USA | Qualitative descriptive design | One-on-one recorded phone interview | To describe women’s  experience of  having  preeclampsia and  being placed on  extended bed rest  during her  pregnancy despite  the newest  recommendations  from ACOG† and  bed rest for  treatment of  preeclampsia | Seven women  who have a  current  diagnosis or  history of  preeclampsia  in the past ten  years through  Facebook  posting | Six themes emerged as  stressors that women  experienced:  Negative feelings and  thoughts; Lack of guidelines  about their diagnosis;  Family stressors; Lack of  social support; Not being  heard; Loss of normal  pregnancy; and Physical  symptoms | 10/10 |
| Mukwenda et al. (2017) | Tanzania | Qualitative | Semi-structured interviews | To explore and  describe women’s  experiences of  having had and  having recovered  from eclampsia at  a tertiary hospital | 10 women who  had eclampsia  admitted to ICU  at a tertiary  hospital | Five main themes were  extracted: Struck  by uncontrollable pain,  seizures and  unconsciousness; Being  cared for and recovered but  unaware of how to  understand and cope with  the disease; Striving against  the troublesome separation  from the baby; Being  connected to God and self-  encouragement; Worrying  over reoccurrence of  eclampsia in future  pregnancies | 10/10 |
| Roberts et al. (2017) | Australia | Qualitative descriptive design | Semi-structured interview | To gain insight into  women's  experience of  hypertension in  pregnancy and to  report on what  mediating factors  may help improve  their experience | 20 women who  were diagnosed  with either  gestational  hypertension  or  preeclampsia  from a regional  　referral  hospital  Interviewed at 10-  12 months  　postpartum | Four main themes were  identified: Reacting to the  diagnosis; Challenges of  being a mother; Processing  and accepting the situation;  Moving on from the  experience.  The mediating factors that  improved the experience  were as follows: Feeling  safe and trusting the care  providers; Having  continuity of care and  career; Valuing social  support from partner,  family and friends | 10/10 |
| Værland et al. (2018) | Norway | Descriptive, qualitative design | Interview (twice) | To describe the  phenomenon of  mothers’  experiences of  being seriously ill  with preeclampsia  and becoming a  mother of a  premature infant | Nine women  experienced  preeclampsia  resulting in  premature  birth from four  hospitals in  different  regions in  Norway | Journeying through physical  and psychological suffering  to be able to care and  assume responsibility for  their beloved infants. The  essence of the journey was  as follows:  Conflicting feelings  concerning giving birth;  Reflecting upon the  borderline of life; Longing  for the infant; Becoming a  mother in actuality;  experiencing physical  exhaustion. | 10/10 |
| Duffy et al. (2019) | UK | Qualitative interview study | In-depth interview | To identify treatment  outcomes relevant  to women with  lived experience of  preeclampsia. | 30 women with  lived  experience of  preeclampsia  within 13 years  between  diagnosis and  interview | Thematic analysis identified 71  different treatment  outcomes. Outcomes that mattered to  women with preeclampsia  were as follows: Pregnancy  and childbirth experience;  Mother's physical health;  Mother's emotional  Health; Baby's physical  health; Child's future  health and well-being. | 10/10 |
| Sandsæter et al. (2019) | Norway | Qualitative research (COREQ) | Focus group interviews | To explore women’s  experiences of  preeclampsia  and/or gestational  diabetes mellitus  (GDM), and their  motivation and  need for  information and  support to achieve  lifestyle changes | 17 women with  preeclampsia  and/or GDM  with a live birth  at a hospital (3-  34 months post  pregnancy) Total of eight  preeclampsia,  moderate  preeclampsia  (n = 3), and  severe  preeclampsia  (n = 5) | Six themes identified:  Trivialization of the  diagnosis during pregnancy;  Left to themselves to look  after their own health; The  need to process the shock  before making lifestyle  changes (severe  preeclampsia); A desire for  information about future,  disease risk and partner  involvement; Practical  solutions in a busy life with  a baby; Healthcare  professionals can reinforce  the turning point. | 10/10 |
| Semasaka et al. (2019) | Rwanda | Qualitative | In-depth interviews | To investigate  women’s  experiences and  perceptions of  specific  complications  during pregnancy  and delivery, and  the consequences  of these  complications on  postpartum health  and family  situation | 15 women with  pregnancy and  delivery-related  complications  from different  districts; Three women  with  preeclampsia  interviewed at  13-24 months  after birth | Identified the theme as  Experiencing challenging  health problems  necessitating reliance on  own resources postpartum.  Four categories contributing  to the theme were  identified as  follows: Being  unknowledgeable and  unprepared for pregnancy  complications; Blaming the  healthcare system;  Developing coping  strategies for a better life;  Hope and fear of the  future. | 10/10 |
| Sripad et al. (2019) | Nigeria | Qualitative | In-depth interviews | To describe the care-  seeking pathways  of Nigerian  women who suffer  from  preeclampsia  and eclampsia. | 42 women who  were survivors  of  preeclampsia  and eclampsia  from seven  states  across  Nigeria | Women’s perceived  susceptibility and threat of  health complications during  pregnancy and childbirth  influence care-seeking  behaviors.  Moderating influences include  acquisition of knowledge,  quality of patient-  provider antenatal care  interactions, and supportive  discussions with families  and communities. These  cues to action mitigate  perceived mobility,  financial, mistrust and  contextual barriers to  seeking timely care. | 10/10 |

Notes:

† American College of Obstetricians and Gynecologist
