# Supplementary material for: Experiences of women with hypertensive disorders of pregnancy: a scoping review
Source: BMC Pregnancy Childbirth. 2022 Feb 22;22:146. doi: 10.1186/s12884-022-04463-y (PMC8864783; doi:10.1186/s12884-022-04463-y)
Supplement: Supplementary file 5 — Additional file 5. [file 12884_2022_4463_MOESM5_ESM.docx]

**Appendix E.** Categories and subcategories of extracted data from the 16 articles analyzed

| Category | 1) Life-threatening disorder | | | 2) Coping with HDP | | | 3) Concerns for baby and challenges of motherhood | | | 4) Fear of recurrence and health problems | | | 5) Necessity of social and spiritual support | | | 6) Positive and negative experiences in the healthcare context | | |
| --- | --- | --- | --- | --- | --- | --- | --- | --- | --- | --- | --- | --- | --- | --- | --- | --- | --- | --- |
| Subcategory | Death is a real possibility | Anguish, blame, and seeking answers | Readiness to accept HDP | Unexpected birth experience | Loss of control | Coping with various physical symptoms | Struggling with the prolonged treatment process for both mother and child | Fears about baby during pregnancy | Emotional roller coaster: Premature baby in NICU | Facing bonding obstacles | Avoiding next pregnancy | Attending to future health issues | Attempting preventive measures | Stronger social networks | Belief in God | Development of trust | Demand for better healthcare | Specific obstacles to healthcare |
| Jackson et al. (2006) |  | ✔ | ✔ |  |  |  |  |  |  |  |  |  |  |  |  | ✔ | ✔ |  |
| Lima de Souza et al. (2007) | ✔ | ✔ | ✔ |  |  |  | ✔ |  | ✔ | ✔ |  |  |  |  |  | ✔ | ✔ |  |
| Barlow et al. (2008) |  | ✔ | ✔ |  |  | ✔ |  |  |  |  |  |  |  | ✔ |  | ✔ | ✔ |  |
| Fleury et al. (2010) |  | ✔ |  | ✔ | ✔ |  |  | ✔ | ✔ | ✔ | ✔ |  |  | ✔ |  | ✔ | ✔ |  |
| de Azevedo et al. (2011) | ✔ | ✔ |  |  |  |  | ✔ | ✔ |  |  | ✔ |  | ✔ |  |  |  | ✔ |  |
| Herbest et al. (2012) | ✔ |  |  |  | ✔ | ✔ | ✔ |  |  |  |  |  |  | ✔ | ✔ |  |  |  |
| Brown et al. (2013) |  | ✔ |  |  |  |  |  |  |  |  | ✔ | ✔ | ✔ | ✔ |  |  | ✔ |  |
| de Souza et al. (2013) | ✔ | ✔ |  |  |  | ✔ |  |  | ✔ |  |  |  |  |  |  | ✔ |  |  |
| Kehler et al. (2016) | ✔ | ✔ |  | ✔ |  | ✔ |  |  |  |  |  |  |  | ✔ | ✔ |  | ✔ |  |
| Mukwenda et al. (2017) |  |  |  |  |  | ✔ |  |  |  | ✔ | ✔ | ✔ | ✔ |  | ✔ | ✔ | ✔ |  |
| Roberts et al. (2017) | ✔ | ✔ | ✔ | ✔ | ✔ |  | ✔ | ✔ |  | ✔ | ✔ |  |  | ✔ |  | ✔ |  |  |
| Værland et al. (2018) | ✔ |  |  | ✔ | ✔ | ✔ | ✔ | ✔ | ✔ | ✔ |  |  |  |  |  |  | ✔ |  |
| Duffy et al. (2019) | ✔ |  |  | ✔ | ✔ | ✔ | ✔ | ✔ | ✔ | ✔ |  | ✔ |  |  |  |  |  |  |
| Sandsæter et al. (2019) |  | ✔ |  | ✔ |  |  | ✔ |  |  | ✔ | ✔ | ✔ | ✔ | ✔ |  |  | ✔ |  |
| Semasaka et al. (2019) | ✔ | ✔ | ✔ |  |  |  | ✔ | ✔ |  |  |  |  |  |  | ✔ |  | ✔ | ✔ |
| Sripad et al. (2019) | ✔ |  | ✔ |  | ✔ |  |  | ✔ |  |  |  |  |  | ✔ | ✔ | ✔ | ✔ | ✔ |
