# Supplementary figures and images for: Experiences of women with hypertensive disorders of pregnancy: a scoping review
Source: BMC Pregnancy Childbirth. 2022 Feb 22;22:146. doi: 10.1186/s12884-022-04463-y (PMC8864783; doi:10.1186/s12884-022-04463-y)

# **Appendix F.** Trajectory of lived experiences of women with HDP within the healthcare context


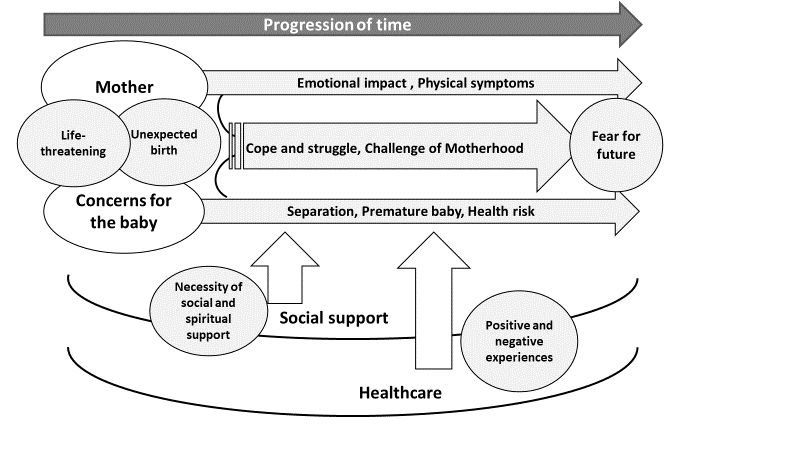

Supplement: Supplementary file 6 — Additional file 6. [file 12884_2022_4463_MOESM6_ESM.docx]
